# Infodemic in Public health a reemerging public health threat: a scoping review

**DOI:** 10.12688/f1000research.130687.1

**Published:** 2023-06-08

**Authors:** Mithun Pai, Shweta Yellapurkar, Aishwarya Shodhan Shetty

**Affiliations:** 1 PUBLIC HEALTH DENTISTRY, MANIPAL COLLEGE OF DENTAL SCIENCES, MANIPAL ACADEMY OF HIGHER EDUCATION (MAHE) MANIPAL, MANGALORE, KARNATAKA, 575001, India; 2 ORAL PATHOLOGY AND MICROBIOLOGY, MANIPAL COLLEGE OF DENTAL SCIENCES, MANIPAL ACADEMY OF HIGHER EDUCATION (MAHE) MANIPAL, MANGALORE, KARNATAKA, 575001, India

**Keywords:** COVID 19, evidence synthesis, infodemics, misinformation, management Public health, social media

## Abstract

Background: Infodemic is a neologism of ‘information’ and ‘epidemic’ coined in the year 2003. Evidence mapping is a technique to appraise the literature which enables the extent of research activity in a specific area to be discovered. The main objective of this evidence synthesis presents the outcomes of an evidence map that was directed to know the extent of Infodemics and its effects on public health.

Methods: The following methods were used to construct this evidence synthesis: Phase I. Construct a Broad Question Referring to the Field of Analysis. Phase II; Defining Key Variables to Be Mapped, identifying the characters of each variable and Outline Inclusion and Exclusion Criteria for the variables. Phase III: Literature search. Phase IV: Screening and Charting the Appropriate Evidence within the Synthesis.

Results:  Authors identified 55 records through database searching, after screening for duplicates, 53 records screened at title/abstract level of which, 16 records were removed because of lack of complete article or articles were not in English. 37 articles were eligible for full text screening, 37 full-text articles were than assessed for eligibility and only 22 articles were included as per inclusion criteria with an interrater Outcome Kappa value: 0.91. The strength of agreement was considered to be 'excellent'.

Conclusions: This synthesis focused majorly on the gaps in the research focused on infodemic. The two main gaps identified were lack of systematically conducted research and poor digital health literacy. As infodemic is a new phenomenon with respect to the COVID-19 pandemic it was an eye opener at different levels of public health, furthermore this evidence map points out areas for further research on the impact of infodemic.

## Introduction

Social media plays a pivotal role in dissemination of information. This information is always not credible, as it is believed to spread misinformation and disinformation. The spread of misinformation is not a new phenomenon but the magnitude of its spread was a concern during the COVID-19 pandemic.

The portmanteau word Infodemic is a neologism of ‘information’ and ‘epidemic’ coined in the year 2003. ^1^ An infodemic means an excessive amount of information of which some are accurate and some inaccurate, usually occurring during a pandemic/epidemic. Similar to the pattern of spread of a pandemic, an infodemic spreads in the same mode, but through digital or/physical information systems, making it more complex for people to isolate a solid and reliable source of information. ^2^^,^^3^

Infodemics have been one of the most virulent phenomena known to humankind, capable of transiting the world instantly. In every possible aspect they mimic a disease, with an epidemiology of its own, symptoms similar to a disease. Sadly it is one of the most highly neglected and underestimated problems. ^4^

The spread of COVID-19 correlated to the spread of misinformation, in terms of circulating conspiracy theories, often dispersed through social media platforms. This constituted a risk to the public and presented a major global health hazard. Delivering a vital source of evidence-based information to the general public during an outbreak of a pandemic aids in quick act of managing the disaster. ^5^

Evidence synthesis is a technique to appraise the literature which enables the extent of research activity in a specific area to be discovered. Systematic reviews usually detail a specific clinical question and seek to answer the question. Evidence mapping on the other hand provides a brief summary of the range, distribution and scope of evidence in a field of interest broadly. ^6^ Evidence synthesis are formulated on an explicit research question concerning the field of interest, which are not in depth analysis of a question but rather a systematic accumulation of topics of interest. ^5^^,^^7^ The evidence synthesis initiates search, and collation of, suitable evidence making use of clear and replicable methods at every step. The synthesis includes clear definition of components, evolution of a detailed and reproducible search strategy, development of clear exclusion and inclusion criteria, and clear conclusions about the level of evidence to be obtained from individual study.

For the present evidence synthesis an open access online tool CADIMA was used for management of evidence that was established by Julius Kühn-Institut (JKI) during a EU-funded project called GMO Risk Assessment and Communication of Evidence (GRACE). CADIMA is a free web tool guiding the conduct and furnishing the documentation of systematic reviews, systematic maps and further literature reviews. ^8^ CADIMA is outlined to offer substantial evidence to users in the structure of prompts, that majorly differentiates between a meticulous systematic review and a quality literature review, this feature substantially reduces the difficulty in assimilation of evidence for new research.

The main objective of this evidence synthesis presents the outcomes of an evidence map that was directed to know the extent of infodemics and its effects on public health during the ongoing pandemic and attempts to explore the nature of evidence present in literature. The process assures a procedure of ‘stocktaking’ of the evidence as vital gateway in providing a sketch of the extensiveness of research activities in the field of infodemics. After consulting experts in Public Health (community medicine), Public Health Dentistry and also the nodal Covid officers of COVID 19, a relevant question was framed and the scope and frame of the was chalked out. The process disclosed two domains, mainly the causes of Infodemics and the effects of infodemics on public health, as the first question was the primary focus of the evidence synthesis two secondary questions were included to widen the scope of this evidence. The objectives of the present review were to screen for good-quality evidence on the effects of infodemics on public health during the Covid-19 pandemic and to know the impact of infodemics and various strategies to manage infodemics in public health during the Covid-19 pandemic.

## Methods

### Eligibility criteria

The authors established a baseline for defining variables for the literature search. The definition of infodemic was established from the World Health Organisation guidelines as ‘infodemic’- is too much information including false or misleading information in digital and physical environments during a disease outbreak’. ^9^ The other key variables included COVID 19 defined as Coronavirus disease (COVID-19) which is an infectious disease caused by the SARS-CoV-2 virus and public health as the topic is novel we included all the available evidence present from Ramdomised control trail, non-randomised controlled trials, systematic reviews, overviews and meta-analyses, as the reviewers wanted to know the range of evidence that would be available for conducting a map for a template for future investigations to be carried out in the field of infodemics. Explanations of the type of review were not definable or are consistently explained as numerous differing terms are used, and many a times interchangeably, therefore only those reviews were included which used a simple methodical search strategy but were systematic in search strategies. There were no restrictions for year of publication but complete articles published in English or with an English translation were included.

### Literature search

The authors designed a search strategy using medical subject headings and specific keywords. There was no restriction on publication date but the language was restricted to English. A search was conducted in two databases namely MEDLINE and Scopus. The reference lists of the included studies were also searched for potential evidence *.*with the following key words Infodemic AND Public AND Health AND Covid-19 NOT Vaccine **.**

The search criteria for MEDLINE and Scopus were devised as they are the two most commonly used databases for medical literature searches. Multiple databases were not screened because this was an evidence synthesis.

The search included the keywords **Infodemic AND Public AND Health AND Covid-19 NOT Vaccine.** Filters were used for Abstract, Clinical Trial, Meta-Analysis, Randomized Controlled Trial, Review, Systematic Review.

### Selection of sources of evidence

Titles and abstracts of all articles deemed important and relevant were recognised by the searches of the two databases. Two reviewers separately selected all the evidence. The search outcomes were screened. Inclusion and exclusion criteria were applied with interrater Outcome Kappa value: 0.91. The strength of agreement was considered to be ‘excellent’.

### Inclusion and exclusion criteria

The reviewers deliberated all studies, regardless of design, as eligible for inclusion. A population and outcome (PO) search strategy that was wide enough to cover all topics was developed for categorizing potentially significant studies in any topic area. We excluded review articles that did not have any systematic search strategies but scrutinized the bibliography for identifying other potentially appropriate evidence. There were no restrictions for year of publication but the articles published in English or with an English translation were included.

The selection of the retrieved articles involved the authors (MP and AS) independently and solved disagreements by consensus or consultation with a third reviewer (SY) Each time the title or abstract reported a keyword of relevance appearing to be eligible for the inclusion, the full article was obtained. All references were retained post the initial screening and later assessed for the inclusion and exclusion criteria the following information was extracted: (i) title, (ii) author, (iii) journal, (v) publication year, (vi) location of key words, (vii) type of article/study and (viii) comments based upon the full texts. all the articles were charted in a standard Excel format. The data extraction sheet can be found under *Underlying data.* ^25^

**Figure 1.**
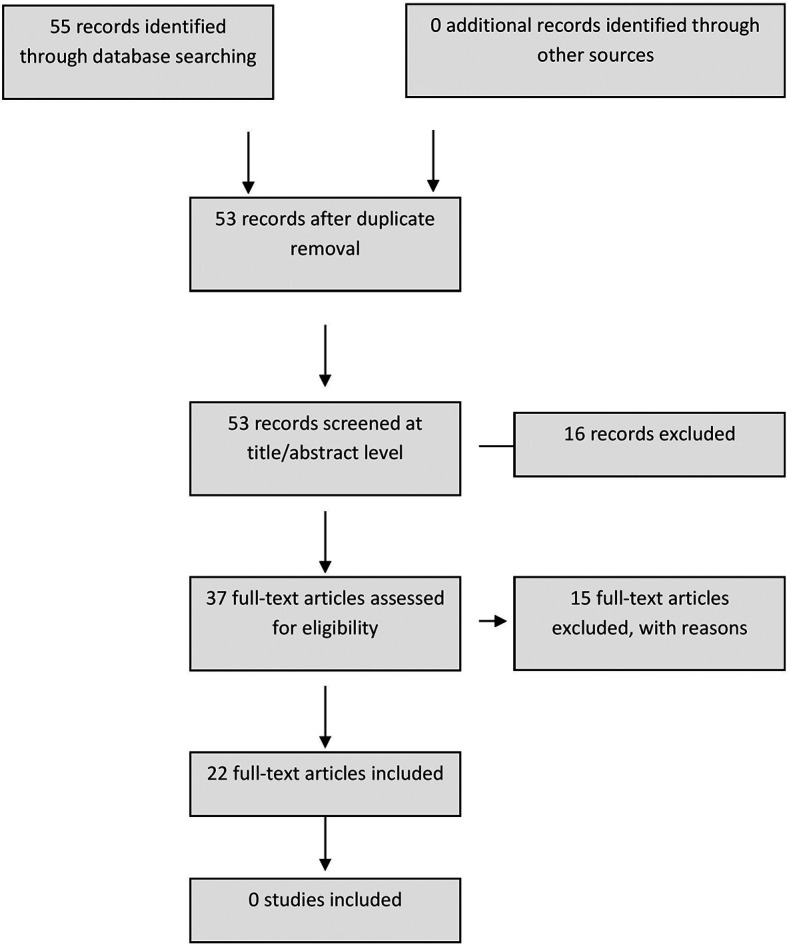
Flow diagram.

The present review drives past the scope of only ‘evidence mapping’ if a systematic review was obtainable than that was inserted into the mapping process and can be considered as an umbrella review and evidence mapping and a brief qualitative explanation of the key results have been considered and each theme is described below ( Figure 1).

## Results

The authors identified 55 records through database searching, after screening for duplicates, 53 records were screened at title/abstract level of which, 16 incomplete or non-English articles were removed and 37 were eligible for full text screening. 37 full-text articles were then assessed for eligibility and only 22 articles were included as per the inclusion criteria with an interrater Outcome Kappa value: 0.91. The strength of agreement was considered to be: ‘excellent’.

The majority of the studies focused on the first domain *impact of infodemic* followed by *effect of infodemic* and *management in infodemic during the covid-19 pandemic.* The studies explicitly mentioned the psychological impacts on infodemics, the health care burden, low digital health literacy, economic burden, financial losses, and desensitization of the public towards infodemics.

### Impact of infodemics *during the covid 19 pandemic*

The studies failed to include some vital aspects such as ways to manage and overcome the infodemic *during the covid 19 pandemic.* A review article by Khan S *et al.* ^10^ and Sharma DC ^4^ elucidated some factors that contributed to the impact of infodemics on health care workers. They found that low immunity due to stress and drop in intellectual capacity due to inadequate resting time led to risk of acquiring infection and psychological traumas. ^11^

Misinformation is ‘false information shared by people who have no intention of misleading others. Disinformation is defined as false information deliberately created and disseminated with malicious intentions.’ ^12^ As this phenomenon spread at a faster rate, health professionals had to come up with a solution and very few health care workers came on social media to clarify this publicly ( Tables 1 and 2). ^13^^,^^14^

**Table 1.** The results of search has been divided into three main headings and the of type evidence under each headings.

Impact of infodemic	Effect of infodemic	Management in infodemic
Systematic review - 2 Narrative review - 6 Cross sectional study -1	Systematic review - 0 Narrative review - 8 Scoping review - 1 Content analysis - 1	Systematic review - 0 Narrative review - 2 Scoping review - 1

**Table 2.** Contains the different themes and synopsis of each article included in the review in detail.

Themes	Author, year	Study type	Synopsis
Impact of infodemic	Khan S *et al.*, 2020	Systematic review	• Psychological disturbances due to pandemic • Improvement in health journalism to improve health literacy • Flaws in conducting research • Faster publication process
	Sharma DC *et al.*, 2020	Narrative review	
	Abbott R *et al.*, 2022	Systematic review	
	Bin Naeem S *et al.*, 2021	Narrative review	
	Dubey S *et al.*, 2020	Narrative review	
	Nowak *et al*., 2021	Narrative review	
	Pian *et al.*, 2021	Narrative review	
	Anwar A *et al.*, 2020	Narrative review	
	Roy D *et al.*, 2021	Narrative review	
Effect of infodemic	Topf J *et al.*, 2021	Narrative review	• Increased publications resulting in bias in peer reviewing process • Anxiety caused by infodemic • Psychological traumas
	Bella E *et al.*, 2021	Narrative review	
	Ying W *et al.*, 2021	Narrative review	
	Delgado *et al.*, 2021	Scoping review	
	Banerjee D *et al.*, 2021	Narrative review	
	Desai A *et al.*, 2022	Narrative review	
	Suarez V J *et al.*, 2022	Narrative review	• due to the uncontrolled circulation of mis information • Digital imbalance of the information • Repercussions of the infodemic on mental health of the elderly • Politicization of the virus
	Gerts D *et al.*, 2021	Content analysis	
	Medford R *et al.*, 2022	Narrative review	
	Casino G *et al.*, 2022	Narrative review	
Management of infodemic	Liu T *et al.*, 2021	Narrative review	• Research area focused on digital health literacy in indigenous populations • Shared decision making (SDM) • Artificial Intelligence (AI) to improve the digital health literacy and to fight infodemics
	Choukou MA *et al.*, 2022	Scoping review	
	Abrama M *et al.*, 2020	Narrative review	

### Effect and management of infodemic during the Covid 19 pandemic

A review by La Bella E ^15^ points out a significant contribution of flaws observed in the systematic reviews that were being published, feeding flawed information to the internet. Bias occurring during peer review and the editorial process has led to the publishing of misinterpreted data to the public. In a cohort of low digital health literates which formed the major portion of the populous, believing deluded information was aphenomenon. ^16^^,^^17^

The existing uncontrolled, unfiltered information provided by the top social media platforms need stricter vigilance, information screening and protection systems. Public awareness of health literacy has to be increased, poor health literacy is also an underestimated global public health problem, and the COVID-19 pandemic has amplified the need for health literacy across the world. Hong *et al.* ^18^ evaluated the students majoring in healthcare on health literacy levels related to COVID-19 infection. Hong *et al.* specified that there was a need to educate and improve the health literacy among the students pursuing health sciences because they form the future of health profession whose responsibility is to educate the masses regarding infodemics if and when there is a future pandemic.

## Discussion

We conducted a synthesis of 55 records from the databases, of which 22 articles were retrieved satisfying the inclusion criteria as predetermined. This amalgamation included 4 phases of systematic data synthesis - *Phase 1* focused on the 3 domains, one - the impact of infodemic on public health, two - its effects on public health and, three – Management of infodemic. Our evidence synthesis revealed that the most effective way to overcome an infodemic phenomenon was to increase digital health literacy by educating the masses and the future research to be directed with an accurate methodological process that is supported by strong evidence.

A review by Choukou *et al.* ^19^ categorised the needs for digital health into five parts. They are 1) knowledge of the disease to be increased by digital health literacy, 2) to manage and cope with new practices and changes from the routine, 3) to overcome anxiety and fear regarding the disease (COVID 19), 4) to overcome the barriers to health literacy and 5) increase the acceptance to technology. ^19^ A study by Li X ^20^ revealed that during the COVID-19 pandemic the main barrier for cohorts with HIV/AIDS to understand their individual health, illness and treatment was considered to be poor health literacy.

Social media as a preventive approach could help in circulating the authentic news and information on diseases. This could help the public in adopting the necessary measures for control of the disease. Lack of digital equipment in vulnerable groups is the basis for poor digital health literacy in population, and resolving this situation through health care organisations reaching out to such groups and enabling access to an electronic source for information and health care of these groups should remain a priority. ^20^

A systematic review conducted to assess the media sources of information and knowledge levels about COVID-19 revealed that 40% of the population depended on social media as their primary source of information regarding the disease. ^21^ The increased circulation of misinformation in social media platforms during the accelerated health emergency has led to a cataclysmal infodemic.

Researchers have the advantage and the right medium to hold back this tide of infodemic and any paucity in conducting the research process could lead to either insufficient information or could be interpreted insufficiently. The rapid spread in false medical cures or false remedies was noticed during the pandemic in large populations. The examples for such false remedies include smelling spices and inhalation of steam with salt could kill COVID 19 before it reached the lower respiratory system. ^22^ Implementing artificial intelligence to tackle this will help publish evidence based and systematically conducted research and filter out the biased publications during a pandemic. ^22^ The quality of research published should be evaluated critically for any misinformation. Hence researchers have to comply with publishing and reporting strong scientific evidence. ^23^

This review has exposed certain definite evidence gaps in the field of infodemics and the ways to manage infodemics during a pandemic. In this unprecedented world with many unexpected situations, infodemics will be a common phenomenon, hence studies on health literacy, and identification of cohorts at high risk of this phenomenon will be a way forward.

### Strength and limitations

Our evidence synthesis was conducted systematically, with inclusion of appropriate literature based on the study objective. The study highlighted the impact and effects of infodemics on public health and ways to manage this phenomenon. The study focused on research gaps in the field of infodemics and has paved way for further research by finding evidence gaps in literature for the same. The major limitation of the study was that we included data from only two databases as the focus was just on finding evidence gaps and exploring further avenues for new research.

## Conclusion

This synthesis focused mostly on the gaps in the field of infodemics and public health. The two main gaps identified were lack of systematically conducted research and poor digital health literacy. As infodemics are a new phenomenon with respect to the COVID-19 pandemic, it was an eye opener at different levels of public health, furthermore this evidence map highlights the need for further research on the impact of infodemics and prevention of their spread. Hence further studies are required to strengthen public health infrastructure and prevent this digital virus.

It has been recommended that the application of Artificial intelligence (AI) in assisting to screen the data available for accurate translation, summarization, simplification and content filtering should be the immediate approach to address this catastrophe.

## Data Availability

Figshare: Infodemic in Public health a reemerging public health threat – evidence synthesis, https://doi.org/10.6084/m9.figshare.21929634. ^24^ This project contains the following underlying data:
-
critical_appraisal_outcome (1).xlsx (the outcomes of each article is critically appraised and described under Article ID, Study ID, Title, Publication year, Authors, Data location, Study name, comments) -
data_extraction_sheet_2021 (1).xlsx (the study for data extraction described under article id, study id, author, publication year, title, data location and study name) Figshare: Infodemic in Public health a reemerging public health threat, https://doi.org/10.6084/m9.figshare.22132904. ^25^
- Flow diagram Data are available under the terms of the Creative Commons Zero “No rights reserved” data waiver (CC0 1.0 Public domain dedication).
