# Supplementary material for: Infodemic in Public health a reemerging public health threat: a scoping review
Source: F1000Res. 2023 Jun 8;12:632. [Version 1] doi: 10.12688/f1000research.130687.1 (PMC10474339; doi:10.12688/f1000research.130687.1)
Supplement: Peer review history

# Peer review: Reviewer response for version 1

**DOI:** 10.5256/f1000research.143459.r194128

**Published:** 2023-09-01

**Authors:** Vijaya Hegde

**Affiliations:** 1 Department of Public Health Dentistry, A. J. Institute of Dental Sciences, Mangalore, Karnataka, India

This is a well-written manuscript that only needs to undergo a few minor changes. This review article focused mostly on the gaps in the field of infodemic and public health. It has recommended the improvement of digital health literacy and use of artificial intelligence as an immediate approach to address this catastrophe.

 The COVID-19 pandemic had massive consequences not only on the societies and health systems across the world, but also has contributed to the development of ‘Infodemic’ to manage the crisis.

 Although one to one consultation between the doctor and the patient remains the gold standard in Medical Care, Infodemic has taken an upper hand during the pandemic. This has enabled simplified access to healthcare, reduction in distance between the patient and doctor, especially where health care is not accessible. However, it also has certain challenges like surplus information from various sources, which may be misinformation or disinformation. This misleads the users, affecting the decision making process thereby delaying health care. Hence an immediate, and collaborative effort is required to  maintain the integrity and credibility of professional expertise and rebuild public trust.

 This is an interesting and unique topic which is well structured and well written. However, I have provided few remarks based on my observation.

**Title:** Kindly specify if the focus is of the impact of COVID-19 related infodemic on Public Health or Infodemic  on Public health.

**Abstract: **The objective could be made more specific as in the manuscript the focus is on COVID -19 related infodemic and its impact on Public Health.

**Introduction:**

- Page 3, L 33-34: Kindly Rephrase the sentence.
- Page 3, L 36: Kindly mention the secondary questions.
- Page 3, L 38-39: Please be more specific.

**Methods:**

- Page 3: Kindly indicate if a review protocol exists the web address where it can be accessed , registration information along with the registration number.
- Page 4: Kindly mention if any attempts were made to confirm the data from the investigators.
- Page 5: Kindly correct the number of studies included in the flow diagram.
- Page 6: Please provide the citations in Table 2.

**Discussions:**

- Page 7: Kindly suggest the other strategies to manage infodemics as mentioned in the introduction **.**
- Page 7: Please address the first domain, i.e the cause of infodemics.
- Page 7: Please consider providing information regarding the management of Psychological disturbance due to the impact of Infodemic.

Are the rationale for, and objectives of, the Systematic Review clearly stated?

Yes

Is the statistical analysis and its interpretation appropriate?

Yes

Are sufficient details of the methods and analysis provided to allow replication by others?

Yes

Are the conclusions drawn adequately supported by the results presented in the review?

Yes

Reviewer Expertise:

Dental Public Health, Bioethics,

I confirm that I have read this submission and believe that I have an appropriate level of expertise to confirm that it is of an acceptable scientific standard.

# Peer review: Reviewer response for version 1

**DOI:** 10.5256/f1000research.143459.r194131

**Published:** 2023-08-31

**Authors:** Shaina Raza

**Affiliations:** 1 University of Toronto, Toronto, Ontario, Canada

The paper is very interesting and well written, the conciseness of the paper is impressive. A few suggestions:

- Talk more about annotators/reviewers biases, their demographics and how authors dealt with that.
- Differentiate between misinformed vs biased information - may see Raza & Ding (2022) ^1^ to support these definitions for citations.
- The contents of table 2 should be mentioned in intro, for clarity (1-2 lines maybe).
- Is 53 and then 27 not a small number for such research (figure 1), also since no time limit is on search strategy, is anything missed? Or so what is the strict criteria?
- How much time did the study take for all this?
- Mention theoretical implication of this study a bit more.

Are the rationale for, and objectives of, the Systematic Review clearly stated?

Yes

Is the statistical analysis and its interpretation appropriate?

Not applicable

Are sufficient details of the methods and analysis provided to allow replication by others?

Yes

Are the conclusions drawn adequately supported by the results presented in the review?

Partly

Reviewer Expertise:

NLP, Informatics, public health, information retrieval

I confirm that I have read this submission and believe that I have an appropriate level of expertise to confirm that it is of an acceptable scientific standard.

## References

[1] : Fake news detection based on news content and social contexts: a transformer-based approach. *International Journal of Data Science and Analytics* .2022;13(4) : 10.1007/s41060-021-00302-z 335-362 10.1007/s41060-021-00302-z PMC880085235128038
